# Preparation and Study of Folate Modified Albumin Targeting Microspheres

**DOI:** 10.1155/2022/3968403

**Published:** 2022-01-28

**Authors:** Quanbin Zha, Ling Zhang, Yuhua Guo, Rui Bao, Feng Shi, Yongkang Shi

**Affiliations:** ^1^Department of Oncology, Jintan Hospital, The Affiliated Hospital of Jiangsu University, Jintan 213200, China; ^2^School of Pharmacy, Jiangsu University, Zhenjiang 212000, China

## Abstract

In this study, folate modified bovine serum albumin was successfully synthesized, while preparation of Nintedanib albumin microspheres (ND-FSA NPs) as a carrier was carried out via electrospinning technology. Folate modified albumin was used to enhance the targeting potential of the prepared microspheres. The prepared microspheres had spherical appearance and smooth outer surface. The diameters of microspheres (764.68 ± 88.46 nm) and zeta potential (- 18.38 ± 0.41 mV) were acceptable. The prepared ND-FSA NPs demonstrated a good degree of modification, wherein the modification rate was 28.1%. *In vitro* release was significantly increased in three different media (double deionized water-DDW, HCl-pH 1.2, and phosphate buffered solution containing 0.5% Tween 80). It is worth noting that incorporation of Nintedanib into folic acid modified albumin microspheres resulted in an enhanced uptake of the drug into MCF-7 breast cancer cells coupled with higher inhibition rate. Altogether, incorporation of Nintedanib into folate modified albumin microspheres is a new approach to improve water solubility and targeting effect of the drug.

## 1. Introduction

Nintedanib (C_31_H_33_N_5_O_4_·C_2_H_6_O_3_S) is a tyrosine kinase inhibitor [1]. Current studies have shown that [2] Nintedanib exerts pleiotropy by inhibiting the activation of fibroblast growth factor (FGF), platelet-derived growth factor (PDGF), vascular endothelial growth factor (VEGF), and Src homologous region 2, including antiangiogenesis and antitumor active domain, like phosphatase-1 (SHP-1). These factors can block signal of pulmonary fibrosis and reduce the proliferation, diffusion, and transformation of fibroblasts, so as to treat idiopathic pulmonary fibrosis (IPF) [3], thereby showing a strong antifibrotic effect, which potentially delays the disease process of IPF and subsequently treats the disease. In the animal model of PF induced by bleomycin or silica particles, Nintedanib could inhibit extracellular matrix (ECM) deposition and reduce the transdifferentiation of fibroblasts into myofibroblasts [4]. In clinical trials, Nintedanib has been used to effectively improve the degree of PF in patients with IPF, wherein it has been approved by FDA to treat this disease [5–8]. Similarly, Nintedanib has been explored in the treatment of some cancer types, namely, cancer of the prostate, kidney, and small lung cells [9]. However, low solubility of Nintedanib in neutral environment coupled with poor absorption by small intestine makes its bioavailability low (only 4.7%) [10, 11]. One of the main causes of intestinal malabsorption of Nintedanib is its pH-dependent solubility, coupled with intestinal mucus, which hinders its absorption [12]. The intestinal solubility of Nintedanib is dependent on pH, wherein its solubility in aqueous solution increases at acidic pH values below 3 but low at neutral pH [13]. In recent times, other workers have tried some nanotechnological approaches to enhance the bioavailability of Nintedanib, such as self-microemulsion delivery system and solid dispersion. However, some areas in Nintedanib nanocarriers development remained unexplored. In particular, there is an unmet need to improve the application of the drug in cancer treatment by enhancing its absorption at the tumor microenvironment (TME).

As a type of globulin in bovine serum (BS), albumin of BS (BSA) is regarded as the most abundant protein in the blood. Various features of BSA make it ideal for targeting hydrophobic drugs like Nintedanib to site of action, namely, biodegradability, high aqueous solubility, high stability, nonimmunogenicity, improved target distribution of tumor microenvironment, high-binding capabilities for drugs, comparatively longer half-life, and minimal toxicity. Besides, it can be combined with a variety of endogenous or exogenous compounds to explore the interaction between serum albumin and anticancer drugs, which may promote improved circulatory stability and delivery of drugs *in vivo*. Hence, it has widely been used in biochemical experiments, especially the fluorescence of BSA, which can be used to monitor drug uptake by cells [14, 15].

Scientific research has found that tumor tissues could capture, accumulate, absorb, and utilize nanoscale molecules such as nanoparticles and liposomes. This phenomenon is called enhanced permeability retention (EPR), which is a significant driver of tumor nanomedicines and can be regarded as a passive targeting process [16–18]. Studies have shown that combination of drugs with BSA can not only improve their targeting but also greatly enhance their water-solubility and other physical and chemical properties, as well as reducing the toxicity of many drugs [19–21]. In comparison with HSA, BSA has wider access and relatively cheap price; therefore, it is more suitable for industrial production and scientific experimental research without special requirements [22].

Albumin nanoparticles can enter tumor tissues by passive targeting [23], or cells via the EPR effect of tumor environment [24]. Indeed, BSA-based nanoparticles have attracted significant attention mainly due to the aforementioned characteristics of BSA, wherein they have been widely used in the investigations of protein binding and applications in targeted delivery of drugs [25, 26]. Meanwhile, as an exogenous molecule of cells, folate has low immunogenicity. It can enter cells in a nondestructive way through special channels, binding to folate receptor (FR), exhibition of high affinity, and further internalization into cells to achieve the purpose of targeting [27]. Moreover, FR is at a low level in normal tissues, but high level is found in malignant cells, such as ovarian, brain, kidney, breast, and bone marrow cancer cells. In this regard, it may be possible to improve the therapeutic effects of anticancer drugs by exploring FR to ascertain the targetability of such drugs. In addition to the advantages mentioned above, folic acid is stable, cheap, and easy to react.

Herein, folate modified albumin was synthesized prior to preparation of folate modified Nintedanib albumin microspheres (ND-FSA NPs). The UV absorption standard curve of Nintedanib was established by UV spectrophotometer. On this basis, the drug loading and entrapment efficiency of folate modified albumin microspheres were evaluated. At the same time, the degree of modifying flu BSA was indirectly ascertained by measuring the content of free amino group of protein by fluorescent amine method. The appearance of the preparation was evaluated through particle size, zeta potential, and scanning electron microscope (SEM) technique, as well as i*n vitro* release, cytotoxicity, and cell uptake experiments.

## 2. Materials and Methods

### 2.1. Materials

EDC, folic acid, and DMSO were bought from Thermo Fisher Scientific. Sulfo-NHS, fluorescent amine, and Rhodamine B were supplied by Shanghai Yuanye Biotech. Co., Ltd. NaHCO_3_, phosphate buffered solution (PBS), and hydrochloric acid (HCl) were provided by Sino-Pharm Chem, reagent Co., Ltd. (Shanghai, China). Aladdin reagent (Shanghai) Co., Ltd., supplied DMF, BSA, PEO, and Nintedanib (purity ≥ 98%), while West Asia Chem. Tech. Co., Ltd. (Shandong, China), provided DMAP. Tween 80 was provided by McLean Biochem. Techn. Co., Ltd. (Shanghai, China). Manufacturing of double deionized water (DDW) was accomplished in-house through Millipore water purification system (Millipore Corp., Bedford, Massachusetts, USA). MCF-7 cells were provided by Shanghai Fuheng Biotechnology Co., Ltd., while other commercially available reagents of analytical grade were obtained for this study.

### 2.2. Establishment of the Nintedanib UV Standard Curve

A certain concentration of Nintedanib solution, folate modified albumin solution, and PEO solution was prepared, respectively. Through scanning within wavelength range of 300∼600 nm, we obtained the UV spectra using appropriate solvent as the reference solution.

An accurately weighed (50 mg) Nintedanib was dissolved in a volumetric flask (100 mL) prior to fixing the volume to obtain the standard solution of Nintedanib, which was detected via dilution to different concentrations (0.000977–0.5 mg/mL). Afterwards, the absorbance of Nintedanib was measured at 450 nm, wherein linear regression was conducted using the concentration (*X*) of Nintedanib with absorbance (*Y*), while two standard curves of different concentrations (high and low) were drawn.

### 2.3. Preparation and Characterization of Folate Modified Albumin

#### 2.3.1. Synthesis of Folate Modified Albumin (FSA)

The EDC (78 mg) and Sulfo-NHS (60 mg) were accurately weighed and dissolved in about 12 mL of anhydrous DMF, while folic acid (108 mg) was weighed and dissolved in about 55 mL of DMSO. Next, the two solutions were mixed, after ice bath amidst stirring for 4 h, and overnight stirring at room temperature to obtain solution A.

Likewise, accurately weighed BSA (400 mg) was fixed to final volume of 30 ml with saturated NaHCO_3_ aqueous solution. Afterwards, solution A was slowly added to BSA solution drop by drop, while 18 mg DMAP was added and stirred continuously for 24 h. Subsequently, 0.2% NaHCO_3_ aqueous solution (3 L) was used as the release medium, which was changed once every 12 h, after 24 h dialysis, while DDW was used for 36 h dialysis, before freeze-drying the substances in the dialysis bag (Mn = 14000 da) for 24 h to obtain the product FSA.

#### 2.3.2. Measurement of Degree of Modification

As mentioned in the previous literature [28], the modification rate of Flu-BSA was indirectly calculated through determination of free amino acids by fluorescent amine method. Fluorescent amine method was also used to ascertain the modification degree of protein indirectly by determining the free amino content of protein.

The specific operations are as follows: 10 mg of BSA and Flu-BSA were appropriately weighed, prior to dissolution in a volumetric flask (10 mL) with appropriate amount of aqueous solution to a constant volume (1 mg/mL) before storage in the refrigerator as stock solution. Later on, 0.1, 0.2, 0.3, 0.4, and 0.5 mL of stock solution (BSA and Flu-BSA) were pipetted into the test tubes, before addition of 1 mL of 0.3% fluorescent aminoacetone after the aqueous solution has been used to top it up to 2 mL, followed by subsequent placement at room temperature for 7 min. The OD value (emission mode, 475 nm emission wavelength, and absorbance at 390 nm) was measured at 390 nm excitation and 475 emission wavelengths of the fluorescence spectrophotometer, while the curve was drawn with OD value as ordinate and concentration as abscissa.

The preparation method of 0.3% (w/v) fluorescent aminoacetone solution was as follows: 30 mg of fluorescamine was weighed accurately into volumetric flask (100 mL), and the volume was fixed to the scale with acetone, kept away from light to 4°C, and stored in refrigerator until use. The calculation formula of degree of substitution (DOS) is as follows:

(1) $$\begin{array}{c} \text{DS}=\frac{1-K_{\text{modified BSA}}}{K_{\text{BSA}}}\times 100\%. \end{array}$$

DS represented the DOS, where respective *K*_modified BSA_ and *K*_BSA_ were the slopes of modified and unmodified BSA.

### 2.4. Preparation of Nintedanib-Loaded Albumin Microspheres

The electrospinning process was carried out based on previous work [29]. Preparation of ND-FSA NPs was carried out through electrospinning technology. Afterwards, FSA (100 mg) and PEO (20 mg) were weighed accurately and 10 mg Nintedanib was added into 10 mL DDW for magnetic rotation stirring. After that, they were evenly dispersed using water bath ultrasound for 30 min.

Then, the working fluid was put into a 10 mL syringe before fixing it on the syringe driver, amidst immobilization of a 23G syringe head on the syringe nozzle, while clamping the clip. Next, the foil was put on the acceptance plate, and the distance between the syringe head and acceptance was controlled at 20 cm, while the speed and the voltage were, respectively, set to 0.12 (1.2 mL/h) and 22 kV to start the machine. After the electrospinning was completed, the powder adsorbed on the foil (ND-FSA NPs) was scraped off with a scraper.

Accordingly, the above procedure was followed only by replacing FSA with BSA, Rhodamine B with Nintedanib, and FSA with BSA to obtain ND-BSA NPs, RB-FSA NPs, and RB-BSA NPs.

### 2.5. Characterization of Nintedanib-Loaded Albumin Microspheres

#### 2.5.1. Determination of Particle Size and Zeta Potential

In order to eliminate interference of buffer, 2 distinct formulations were dispersed in 10 mL DDW. Using techniques like dynamic light-scattering (DLS) and phase analysis light-scattering (PALS), we determined the particle sizes and zeta potentials of ND-BSA NPS and ND-FSA NPS with Nano-Brook 90 plus pals (Brookhaven Inst. Corp., Holtsville, NY, USA). Measurements were done in triplicate at scattering angle of 90° and temperature of 25°C.

#### 2.5.2. SEM Analysis

We evaluated surface morphology, shape, and size of ND-FSA NPs via SEM (Tescan electron microscope Vega-TSS 130 mm). Prior to observation, we mounted the samples on metal holders and vacuum coated with gold-palladium layer.

#### 2.5.3. Estimation of Entrapment Efficiency (EE) and Drug Loading (DL)

The EE and DL of ND-FSA-NPs were determined using membrane method. Two parts of ND-FSA NPs (5 mg) were measured accurately, with one part being added to 4 mL methanol solution, before it was demulsified with water bath ultrasonic for 20 min after eddying for 10 s, and 10000 rpm centrifugation for 5 min. Later on, supernatant was taken to determine the concentration of Nintedanib by UV, with the total ND mass in ND-FSA NPs (5 mg) calculated ultimately. The other part was added to 2 mL DDW, and the ND-FSA NPs were removed through 0.45 *μ*m water filtration membrane after vortexing for 10 s. The concentration of free ND-FSA NPs was measured by UV, while the mass of the free ND-FSA NPs was calculated. A triplicate experiment was performed in parallel. The following formula was used to calculate the EE and DL:

(2) $$\begin{array}{c} \text{EE}\left(\%\right)=\frac{w_{\text{total}}-w_{\text{free}}}{w_{\text{total}}},\\ \text{DL}\left(\%\right)=\frac{w_{\text{total}}-w_{\text{free}}}{w_{\text{all}}}, \end{array}$$

where *W*_total_ represented the mass of Nintedanib measured after methanol demulsification of microspheres and *W*_free_ represented the free drug amount, while *W*_all_ indicated the mass of ND-DADPI-NPS microspheres (5 mg).

### 2.6. *In Vitro* Release

Dialysis method was used to ascertain the *in vitro* release pattern of ND-FAS NPs. Preparation of Nintedanib suspension was carried out prior to this experiment by dissolving the native drug (5 mg) in 1 mL of 3 dissolution media, namely, HCl solution (pH1.2), DDW, and PBS with 0.5% Tween 80 via ultrasonication technique. Afterwards, the dialysis bags (Mw = 3,500, 25 mm × 5 mm, Green Bird Sci. and Tech. Dev., Shanghai, China) were filled with free Nintedanib (1.0 mL each, 5 mg/mL) and ND-FSA NPs (1.0 mL, 5 mg/mL). Next, ends of the dialysis bags were sealed before they were immersed in the aforementioned release media (100 mL) amidst 24 h storage in single-neck flask at 37 ± 0.5°C under continuous shaking at 100 rpm speed using constant temperature water bath vibrator (SHZ-88, Jintan Medical Inst. Corp., China). At predesigned intervals (0.083, 0.25, 0.5, 0.75, 1, 2, 3, 4, 6, 8, 10, 12, and 24 h), we sampled 1.0 mL of the dissolution media and rapidly refreshed with equal amount of the media. Using cellulose nitrate membrane (0.22 µm), we filtered samples of each time point prior to the determination of absorbance via method stated in Section 2.2. Of note, all triplicate assays were performed.

### 2.7. Cytotoxicity Testing with MTT Assay

The MCF-7 cells in logarithmic growth stage were treated with 40000 cells/mL, while 100 *μ*L/per well cells were inoculated in a 96-well plate cell incubator for culturing. Next, varied concentrations (5, 25, 100, and 150 *μ*g/L) of ND-FSA NPS solution or non-drug-loaded blank carrier (FSA NPs, the corresponding ND-FSA NPS (50, 100, 200, 300, and 400 *μ*g/mL)) were used to separately treat the cells after 24 h inoculation, wherein the cells were washed (twice) with PBS after 24 h incubation and then washed twice with PBS buffer. Culturing of the cells was continued for 4 h after addition of MTT solution (20 *μ*L, 5 mg/mL) per well. Subsequently, MTT solution was removed before addition of DMSO (100 *μ*L) to each well, after shaking the decolorization shaker for 10 min. Next, the absorbance (OD) value of each hole was detected by enzyme labeling instrument, using an absorption wavelength of 570 nm. The half inhibitory concentration IC_50_ of each group was calculated using an IBM SPSS software. Meanwhile, the cell survival rate of each group was calculated according to the formula as follows:

(3) $$\begin{array}{c} \text{cell survival rate}=\frac{{\text{OD}}_{T}}{{\text{OD}}_{c}}\times 100\%. \end{array}$$

### 2.8. Study of Cellular Uptake

Rhodamine dyes are usually used as fluorescent probes because of their high coefficient of absorption and broad fluorescence in the visible region of electromagnetic spectrum [30]. Therefore, Nintedanib is replaced by the fluorescent probe Rhodamine B in this part, and the RB-BSA NPs and RB-FSA NPs coated with Rhodamine B were prepared according to the preparation process of ND-Flu-BSA.

The MCF-7 cells in logarithmic growth stage were inoculated on a six-well plate (1 ×10^5^/mL, 2 mL), before discarding the culture medium after RAW264.7 has adhered to the wall. Next, RB-BSA NPs and Rb FSA NPs solution containing the same Rhodamine B were added to the cells before 1.5 h incubation and washing (thrice) with PBS buffer. Later on, the cells were fixed with 4% paraformaldehyde (1 mL) for 20 min and rinsed (thrice) with PBS, while the nucleus was dyed with 0.5 mL DAPI. Afterwards, the cells were incubated at 37°C for 20 min and washed with PBS (pH 7.4) 3 times, and one drop (50 *μ*L) of antifluorescence quenching sealing solution was added on the sample with 1 mL pipette. The antifluorescence quenching sealing solution was placed on the sample. Finally, the fluorescence inverted microscope was observed with blue light (DAPI labeled nucleus) and red light (Rhodamine B).

## 3. Results and Discussion

### 3.1. Establishment of the Nintedanib UV Standard Curve

**Figure 1:**
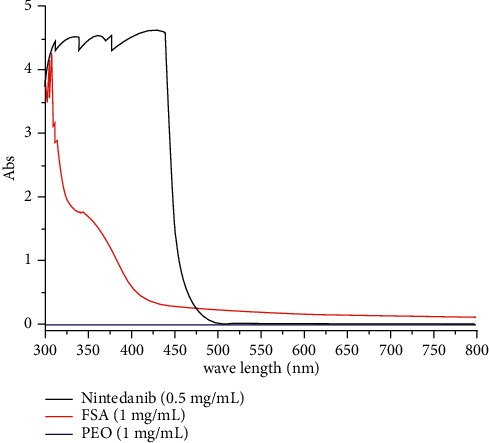
UV full wavelength scanning spectrum. (a) Nintedanib, (b) folate modified albumin, and (c) PEO.

The UV absorption of the three substances was determined by UV spectrophotometric technique. As shown in Figure 1, PEO did not show obvious UV absorption within the detection wavelength, while folate modified albumin showed a certain intensity of UV absorption, but they were all within the range of 300∼400 nm. The Nintedanib solution showed strong UV absorption at lower wavelength less than 425 nm, but the UV absorption decreased with an increase in the detection wavelength. However, in order to avoid the influence of folate modified albumin, 450 nm was finally selected as the detection wavelength of Nintedanib, wherein the standard curve was established, while subsequent content determination was carried out.

Two standard curves of high and low concentrations were established by taking absorbance as ordinate and concentration of Nintedanib standard solution as abscissa. The accompanying linear regression equation was *Y*_1_ = 2.3632*X* + 0.0013 (*n* = 5, *R*^2^ = 0.9991, linear ranged 0.000977–0.015625 mg/mL) and *Y*_2_ = 2.9556x - 0.0383 (*n* = 5, *R*^2^ = 0.9991, linear ranged 0.03125–0.5 mg/mL). After verification of recovery, precision, and repeatability of standard drugs, we observed that the differences were within satisfactory limits.

### 3.2. Establishment of UV Standard Curves of BSA and FSA and the Calculation of Modification Degree

**Figure 2:**
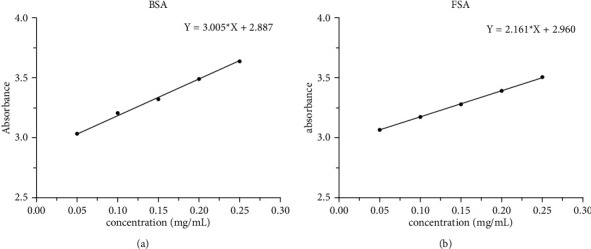
The standard curves of BSA (a) and FSA (b).

The absorbance of BSA and FSA at 390 nm was measured by fluorescence spectrophotometer with the emission wavelength of 475 nm. The standard curves of BSA and FSA were drawn with OD value as ordinate and concentration as abscissa, wherein the respective equations were *Y*_1_ = 3.0054*X*_1_ + 2.8871 (*R*^2^ = 0.9965, *n* = 3) and *Y*_2_ = 2.161*X*_1_ + 2.9596 (*R*^2^ = 0.9996, *n* = 3). The established standard curve was linear (Figure 2). According to the slope of the two standard curves and the corresponding modification formula, the modification rate of FSA was estimated to be 28.1%.

According to the rheological mechanical properties [31], the greater the degree of modification, the closer the three-dimensional structure of albumin microspheres, while the more complex the spatial network structure, the greater the mechanical strength. In terms of antienzymolysis ability, the greater the degree of modification, the lesser the enzyme binding domain in the molecular structure of the microspheres, while stronger antienzymolysis ability results in longer half-life *in vivo*. Also, in terms of biocompatibility, the greater the degree of modification, the greater the structural change of the preparation, which could result in easy rejection by the body and reduction of biocompatibility. Altogether, the degree of modification determined many properties of albumin microspheres, wherein it was closely related to its application value.

### 3.3. Evaluation of Nintedanib-Loaded Albumin Microspheres

#### 3.3.1. Particle Size and Zeta Potential

**Figure 3:**
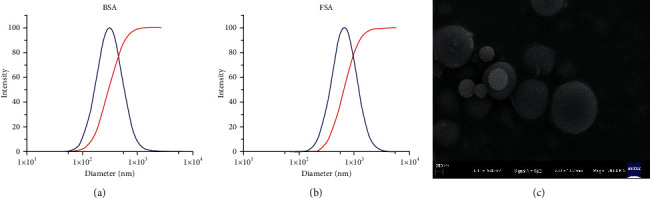
Particle size distribution of ND-BSA NPS (a) and ND-FSA NPS (b). Scanning electron micrographs of ND-FSA NPS (c).

Calculation of the mean diameter of the 2 different microspheres was performed using their individual particle sizes. As shown in Figures 3(a) and 3(b), the average particle size for ND-BSA NPS and ND-FSA NPS was 316.47 ± 30.25 and 764.68 ± 88.46, respectively. Usually, the rate of drug release and the degree of release are greatly affected by particle size of nanoparticles, wherein it has significant impact on rate of drug absorption in gastrointestinal tract (GIT) and *in vivo* availability. SEM micrographs of ND-FSA NPS (powder form) are displayed in Figure 3(c). Through the images (Figure 3(c)), we observed the spherical shape of ND-FSA-NPS coupled with uniform distribution of particle size (roughly 760 nm) and smooth outer surface, which matches the results of DLS. Internal structural examination of microspheres displayed solid interior architecture with no pores or perforation. Likewise, the potential measurement results showed that the two kinds of microspheres had higher negative potential, which were -31.39 ± 1.45 mV and -18.38 ± 0.41 mV, respectively. It has been established that low zeta potential results in higher repulsive force between particles, whereas stable formulation has greater transmembrane of the drug [32, 33].

#### 3.3.2. EE and DL of ND-DADPI-NPs

Accordingly, the EE of ND-DADPI-NPS and DL of Nintedanib in ND-DADPI-NPS were determined. Based on the standard curve (*Y*_2_ = 2.9556*x* − 0.0383, *R*^2^ = 0.9991), we determined the DL of ND-DADPI-NPS to be 3.92 ± 0.19%, while EE was 87.46 ± 0.66%.

### 3.4. *In Vitro* Release Pattern of ND-FSA NPs

**Figure 4:**
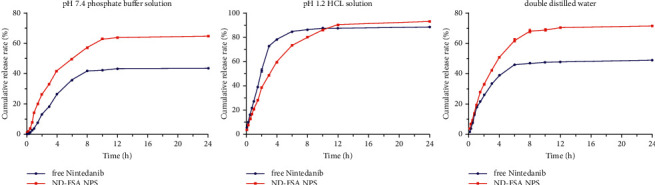
Profiles of *in vitro* release of free Nintedanib and ND-FSA NPS (5 mg/mL for each) in 3 dissolution media (100 mL), namely, HCl solution (pH 1.2) (a), phosphate buffered solution (PBS) with 0.5% Tween 80 (pH 7.4) (b), and double deionized water (DDW) (c), at distinct time points (within 24 h mean ± SD, *n* = 3).

Dialysis method was employed to investigate release patterns of Nintedanib from microsphere and free drug.  Figure 4 shows cumulative *in vitro* release profiles of ND-FSA NPs in 3 media. Faster release rate was observed for Nintedanib-loaded microsphere compared to free drug in PBS (pH7.4) with 0.5% Tween 80 and DDW. From Figure 4, higher release rate of Nintedanib from microsphere was observed in PBS (64.49%) in comparison with free drug suspension (43.29%) within 24 h. Likewise, in DDW, 70.87% of Nintedanib was released from microsphere within 24 h compared to 48.42% from free drug suspension. During the first 10 h, the release rate of Nintedanib in the microsphere preparation in pH1.2 HCl was slower than that of free drug, which was different from that in PBS and DDW. However, the cumulative release rate of Nintedanib in the preparation maintained the same trend as that in the other two release media. In particular, 92.17% of Nintedanib was released from ND-FSA NPS in DDW compared to 87.72% from free drug. It can be inferred that Nintedanib dissolution improved substantially after its incorporation into folate modified albumin microspheres carrier, which is supported by other related literature studies [34]. Available literature suggests that albumin and folate are very soluble in water, which may have contributed to the improved release rate of Nintedanib in DDW. Additionally, we observed different release patterns for ND-FSA NPs and the free drug in the pH gradient media.

The release trend of Nintedanib-loaded microsphere was comparatively slow in pH 7.4 comparable to other media, and it was related to the low solubility of Nintedanib in pH7.4 PBS [9], again, which may suggest minimum initial burst release in the GIT. The prepared microspheres may have a certain sustained-release effect [35]. This may be due to BSA, which being a protein macromolecule can shuttle out only when the microsphere gap is large enough. Thus, when the microsphere gap is large enough, the release rate of BSA-based drugs was increased. This is because the volume of the microsphere swells rapidly and the pore increases with time. Therefore, the number of molecules that BSA can shuttle increases to the highest point [36]. Importantly, Nintedanib might be slowly delivered to the site of increased drug absorption, probably owing to the release features of ND-FSA NPs in the GIT environment. Thus, the oral *in vivo* availability of Nintedanib can be potentially enhanced by the folate modified albumin targeting microsphere carrier.

### 3.5. Result of the Cytotoxicity Test

**Figure 5:**
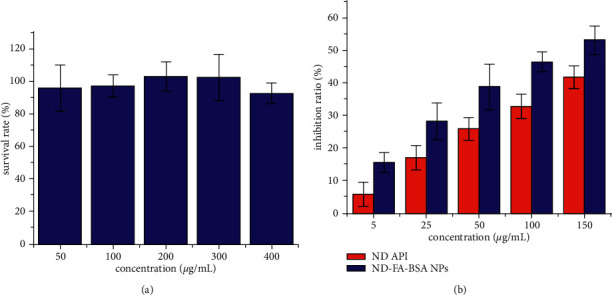
Survival rate of MCF-7 cells by carrier materials (a) and inhibition rate of ND API and ND-FSA NPs on MCF-7 cells (b); mean ± SD, *n* = 3.

It can be observed from Figure 5(a) that when the concentration of blank carrier FSA NPs was in the range of 50–400 *μ*g/mL, there was a slight difference in the survival rate of MCF-7 cells, but they were all more than 90%. It was proven that the carrier material has no toxicity or only minimal toxicity to cells and did not have any meaningful impact on the subsequent experimental results.

After MCF-7 cells were treated with different concentrations of ND API and ND-FSA NPs for 28 h, the absorbance values of each well were measured by microplate reader at 570 nm wavelength, while the average value was calculated. Results as shown in Figure 5(b) depicted that an increased concentration of ND API and ND-FSA NPs resulted in higher inhibition rate on MCF-7 cells. Notably, we could easily observe that the inhibition rate of ND-FSA NPs on MCF-7 cells was significantly higher than that of ND API at the corresponding concentration. This shows that after Nintedanib was incorporated into folate modified albumin microspheres, its inhibitory effect on cancer cells was enhanced under the same *in vitro* conditions. To a certain extent, we may consider that Nintedanib-loaded microsphere could effectively release more therapeutic factors, which matched the *in vitro* release results.

**Figure 6:**
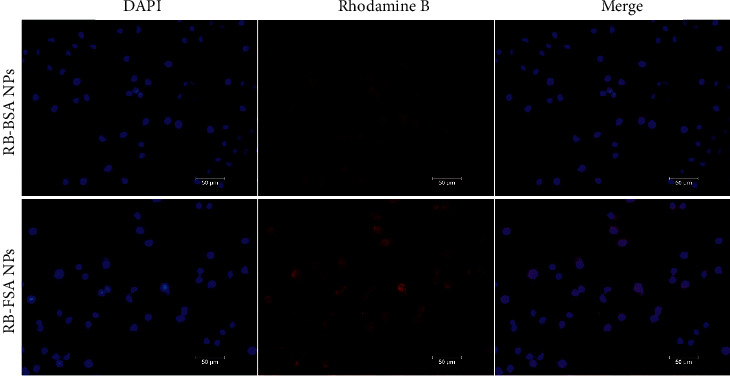
Drug uptake capacity of MCF-7.

In order to further explore whether MCF-7 has an influence on Nintedanib uptake, Rhodamine B was used as a fluorescent probe instead of Nintedanib to prepare two kinds of encapsulated Rhodamine B microspheres with or without folate modification. After treating MCF-7 cells with RB-BSA NPs and Rb FSA NPs that contained the same content of Rhodamine B, we observed the fluorescence intensity to further determine the drug uptake ability of the cells. As shown in Figure 6, the fluorescence intensity of Rhodamine B microsphere preparation modified with folic acid was significantly higher than that of unmodified Rhodamine B preparation. This suggests that microspheres modified with folic acid could enter MCF cells more, thereby obtaining greater uptake capacity and demonstrating a better therapeutic effect.

## 4. Conclusion

In this study, folate modified albumin (FSA) was successfully synthesized prior to the preparation of Nintedanib folate modified albumin microspheres (ND-FSA NPs). Physical characterization of ND-FSA NPs showed that ND-FSA NPs were spherically shaped, in addition to the uniform distribution of particle size, smooth outer surfaces, nanoscale particle size, and low potential, as well as higher EE (about 87%). *In vitro* dissolution test indicated that solubility of Nintedanib increased substantially in DDW. Also, it was observed that ND-FSA NPs had a good degree of modification, which could improve the half-life of the drug to a certain extent. In addition, cytotoxicity and cell uptake experiments showed that, after encapsulation of Nintedanib into folic acid modified albumin microspheres, the cell uptake ability of the drug was significantly enhanced, while the inhibition rate of breast cancer cell MCF-7 was higher. In general, this study may provide support for potential targeted delivery of Nintedanib into tumor cells.

## Data Availability

The data presented in this study are available on request from the corresponding author.
